# Supplementary material for: Polyoxometalate-Decorated Gold Nanoparticles Inhibit β-Amyloid Aggregation and Cross the Blood–Brain Barrier in a µphysiological Model
Source: Nanomaterials (Basel). 2023 Oct 3;13(19):2697. doi: 10.3390/nano13192697 (PMC10574493; doi:10.3390/nano13192697)
Supplement: Supplementary file 1 [file nanomaterials-13-02697-s001.zip › nanomaterials-2637243-supplementary.pdf]

nanomaterials
MDPI

Supplementary Materials

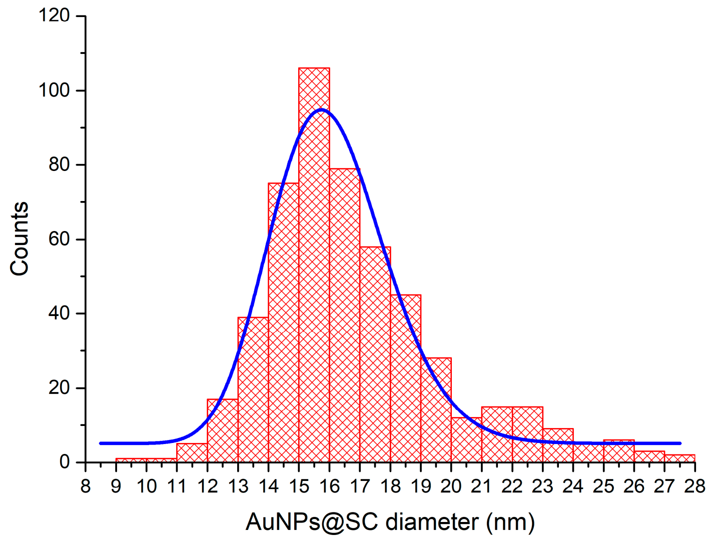

Figure S1. Histogram of citrate capped gold nanoparticles (AuNPs@SC), fitted to a LogNormal distribution with the following parameters: Center = 15.95 nm, offset = 5,06 and log standard deviation = 0.11 nm. The size distribution was obtained by measuring the size of n = 522 particles from three different synthesis batches in Bright Field Scanning Transmission Electron Microscopy (BF-STEM) images.

Table S1. IR peaks of different compounds synthetized compared in the last two columns with the literature.

AuNPs@ POM	AuNPs@POM@PEG	POM	POM [1,2]	PEG [3]
	1350.2			1343
	1298.1			1279
	1249.9			1240
	1137.5			1130
	1095.6			1094
987.6	987.6	989,5	988	
943.2	941.3	943,2	945	951
869.9	868.0	872,9	875	
858.3	852.5	852,5	855	840
798.5	796.6	790,8	805	
731.0	727.2	713,7	730	
513.1	509.2	505,6		

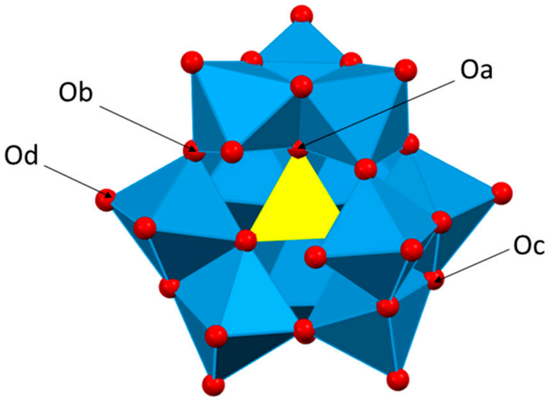

Scheme 1. Structure of the β2 isomer of the monolacunary Keggin ion $[SiW_{11}O_{39}]^{-8}$ with labelling of the different oxygen atoms. WO_6 octahedra (blue) and SiO_4 tetrahedra (yellow). Adapted from (Chermette and Lefebvre, 2012). [4]

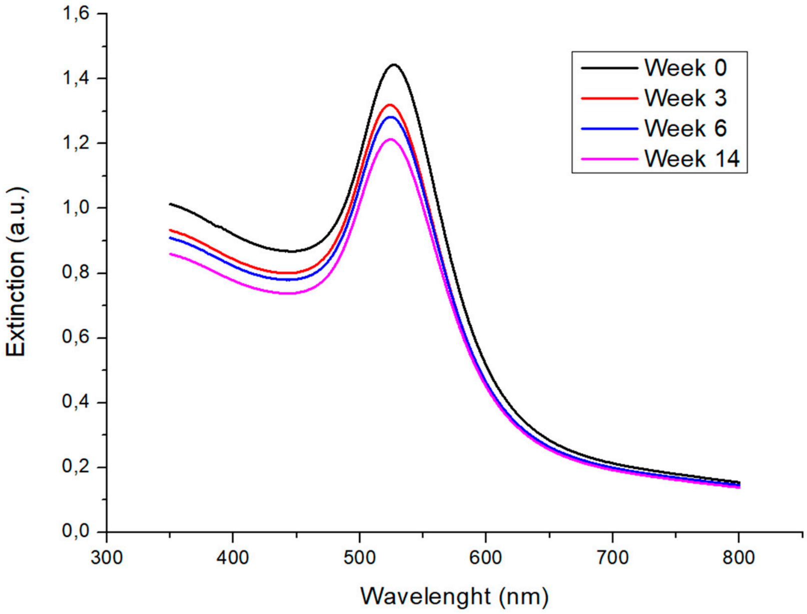

Figure S2. Non normalized UV-Vis spectra of AuNPs@POM@PEG in PBS after up to 14 weeks, showing particle long-term stability.

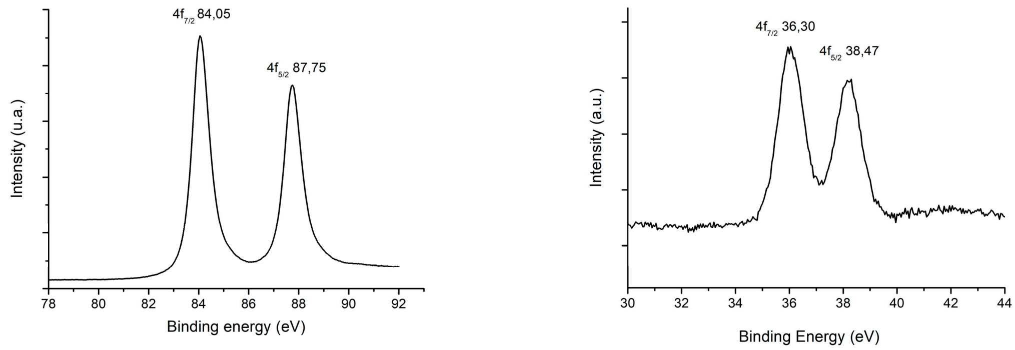

Figure S3. XPS spectra of AuNPs@POM. On the left, Au peaks. On the right, W peaks.

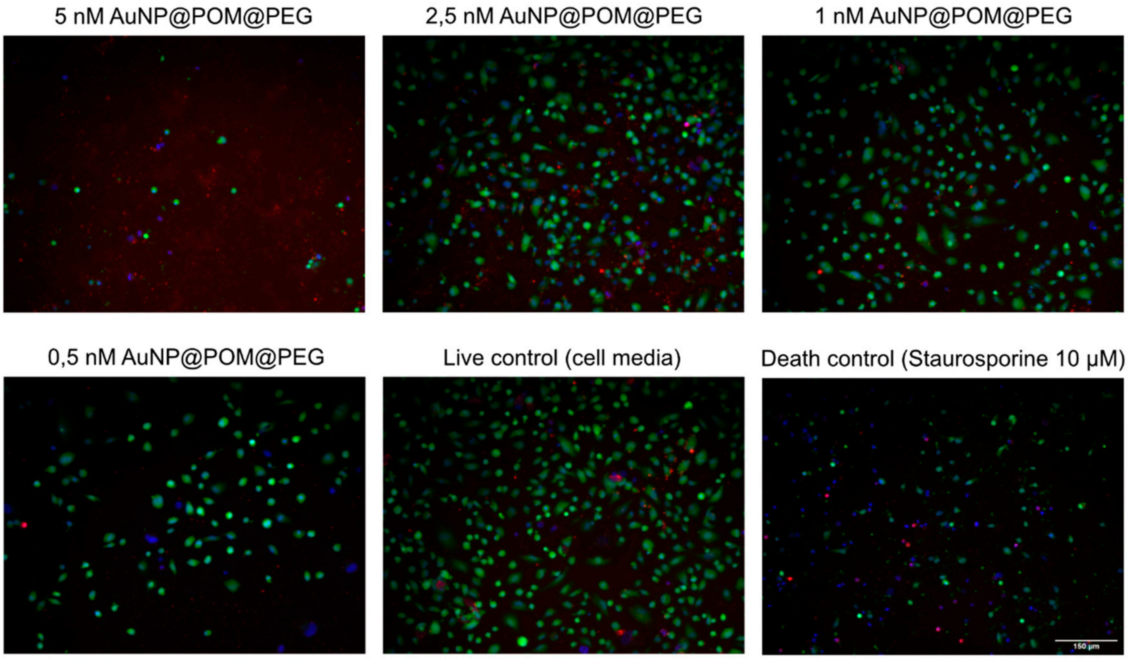

Figure S4. Representative fluorescence images of the Live/Dead assay obtained for endothelial cells under different treatment conditions. Green, red and blue signal indicate alive, death cells and cell nuclei, respectively.

References

1. A. Tézé and G. Hervé, Journal of Inorganic and Nuclear Chemistry, 1977, 39, 999–1002.
2. Alvin P. Ginsberg, Ed., Inorganic Syntheses, John Wiley & Sons Ltd, New York, 1991.
3. K. Shameli, M. Bin Ahmad, S. D. Jazayeri, S. Sedaghat, P. Shabanzadeh, H. Jahangirian, M. Mahdavi and Y. Abdollahi, Int J Mol Sci, 2012, 13, 6639–6650.
4. H. Chermette and F. Lefebvre, Comptes Rendus Chimie, 2012, 15, 143–151.
